# Long‐term follow‐up of a young male who developed acute macular neuroretinopathy following COVID‐19 vaccination

**DOI:** 10.1002/ccr3.8181

**Published:** 2023-11-14

**Authors:** Shunsuke Ikema, Gen Miura, Daisuke Shimizu, Takayuki Baba

**Affiliations:** ^1^ Department of Ophthalmology and Visual Science Chiba University Graduate School of Medicine Chiba Japan

**Keywords:** acute macular neuroretinopathy, coronavirus, COVID‐19, vaccine

## Abstract

This report presents the clinical findings and prognosis of a healthy male patient who developed acute macular neuroretinopathy after COVID‐19 vaccination. Abnormal findings improved about 1 month after the onset and disappeared 3 months later. The subjective symptoms disappeared in 3 months, and no recurrence was observed for 1 year.

## 1 BACKGROUND

Several cases of patients who developed various ocular complications following coronavirus disease 2019 (COVID‐19) infection and vaccination have been reported. Specifically, cases of patients who developed herpes zoster ophthalmicus,^1^, ^2^, ^3^ uveitis in Vogt‐Koyanagi‐Harada disease,^4^, ^5^, ^6^, ^7^, ^8^ multifocal choroiditis,^9^ scleritis,^10^ multiple evanescent white dot syndrome,^11^, ^12^ acute posterior multifocal placoid pigment epitheliopathy,^4^ acute zonal occult outer retinopathy,^4^, ^13^ optic neuritis,^14^, ^15^, ^16^, ^17^, ^18^ ischemic optic neuropathy/optic nerve infarction,^13^, ^19^, ^20^ corneal graft rejection,^21^ and Bell's palsy^22^ after COVID‐19 have been reported. Development of other retinal disorders after COVID‐19 infection and vaccination have also been reported, with acute macular neuroretinopathy (AMN) and paracentral acute middle maculopathy (PAMM) being particularly common. AMN is believed to be caused by disruption of the deep retinal capillary plexus owing to an ischemic event and is suggested to be associated with nonspecific flu‐like illness or fever and the use of contraceptives.^23^, ^24^ Most patients with AMN (>80%) are female.^23^, ^24^ Only a few cases of male patients who developed AMN after COVID‐19 have been reported. To our knowledge, no long‐term clinical course of a male patient who developed AMN after COVID‐19 vaccination has been reported to date. In this report, we present the findings of the long‐term follow‐up of a male patient who developed AMN following COVID‐19 vaccination.

## 2 CASE PRESENTATION

### 2.1 Clinical findings 3 days after COVID‐19 vaccination

A 19‐year‐old Asian male was referred to our department for ophthalmologic examination because of sudden onset of blurred vision in his left eye 2 days after receiving the second dose of the Moderna SARS‐CoV‐2 mRNA vaccine. The day after the vaccination, he developed a fever of 39.7°C and a headache. Three days after the vaccination, he visited a doctor who observed macular discoloration and disruption of the macular ellipsoid zone (EZ) on optical coherence tomography (OCT); therefore, he was referred to our hospital. He visited our hospital 9 days after the vaccination. He did not receive any treatment from the previous physician. He reported no history of systemic and ocular disease.

**FIGURE 1:**
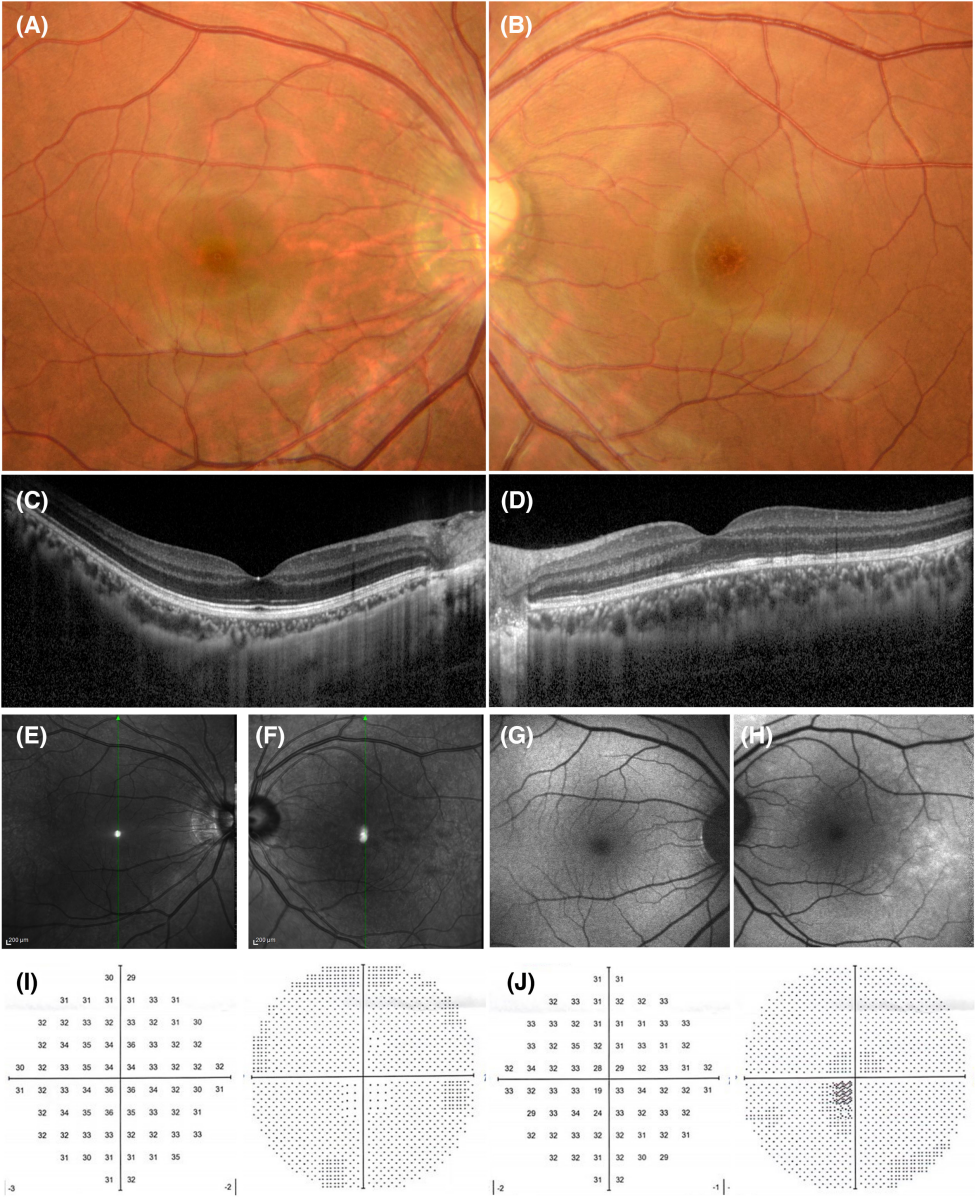
Fundus photographs (A, B) and optic coherence tomography (C, D), near‐infrared reflectance (E, F), fundus autofluorescence (G, H), and 10‐2 visual field test (Humphrey field analyzer) (I, J) findings at 3 days after the vaccination.

Laboratory investigations showed that his complete blood count and serum alanine transaminase, aspartate transaminase, alkaline phosphatase, creatinine, blood urea nitrogen, and C‐reactive protein levels were within normal limits. Initial ophthalmological examination showed that his best‐corrected visual acuity (BCVA) was 20/16 in both eyes. The intraocular pressure of his right eye and left eye was 12 mmHg and 10 mmHg, respectively. His pupillary light reflex was normal, and no relative afferent pupillary defect was detected in either eye. Slit‐lamp examination of the anterior segments and lenses of both eyes revealed normal findings. However, bilateral fundoscopy revealed a clear vitreous and a small yellowish‐white patchy discoloration of the fovea in the left eye. (Figure 1B). OCT revealed a discontinuity in the fovea and temporal ellipsoid zone of the left eye. The choroidal thickness of the eye was within normal limits (Figure 1D). Near‐infrared reflectance (NIR) imaging of the left eye revealed multiple patchy hyporeflective lesions that involved the fovea and extended temporally (Figure 1F). Fundus autofluorescence (FAF) showed hyperfluorescence in the area corresponding to the hyporeflective lesions on NIR (Figure 1H). Visual field analysis performed using the Humphrey field analyzer (HFA) 10‐2 program showed loss of sensitivity in the center of the left eye (Figure 1J). All the examination findings for the right eye were normal (Figure 1A, C, E, G, I). Based on these findings, this patient was diagnosed with AMN of the left eye.

### 2.2 Clinical findings 30 days after the vaccination

**FIGURE 2:**
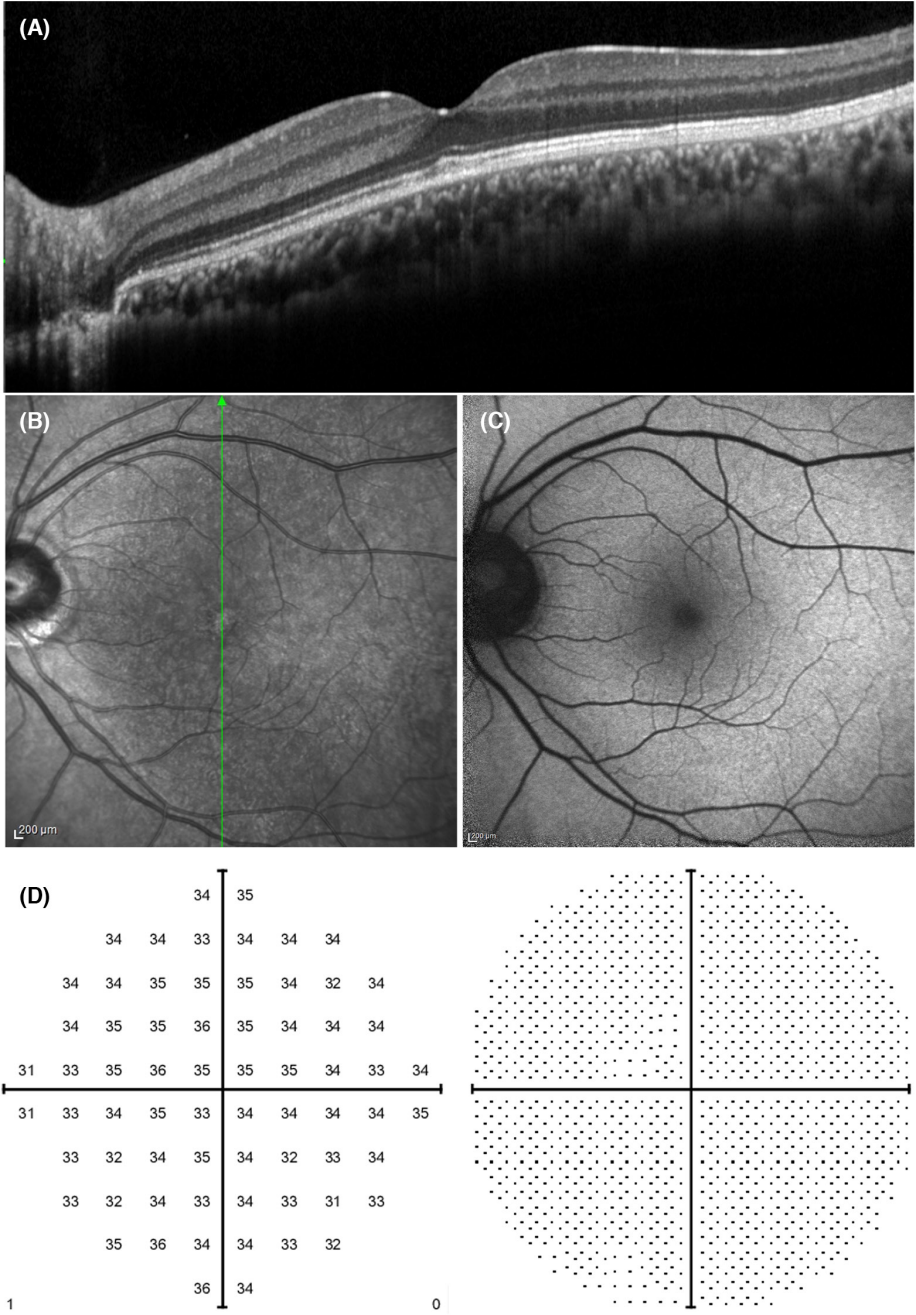
Optic coherence tomography (A), near‐infrared reflectance (B), fundus autofluorescence (C), and 10‐2 visual field test (Humphrey field analyzer) (D) findings of the left eye at 30 days after the vaccination.

The patient's BCVA was 20/16 in both eyes. The blurred vision in his left eye did not improve even 1 month after the vaccination. However, OCT showed that the discontinuity in the fovea and temporal ellipsoid zone of the left eye had improved (Figure 2A). In addition, the hyporeflective lesions and hyperfluorescence observed on NIR and FAF, respectively, had improved (Figure 2B,C). The loss of central field sensitivity in the left eye improved as well (Figure 2D).

### 2.3 Clinical findings 86 days after the vaccination

**FIGURE 3:**
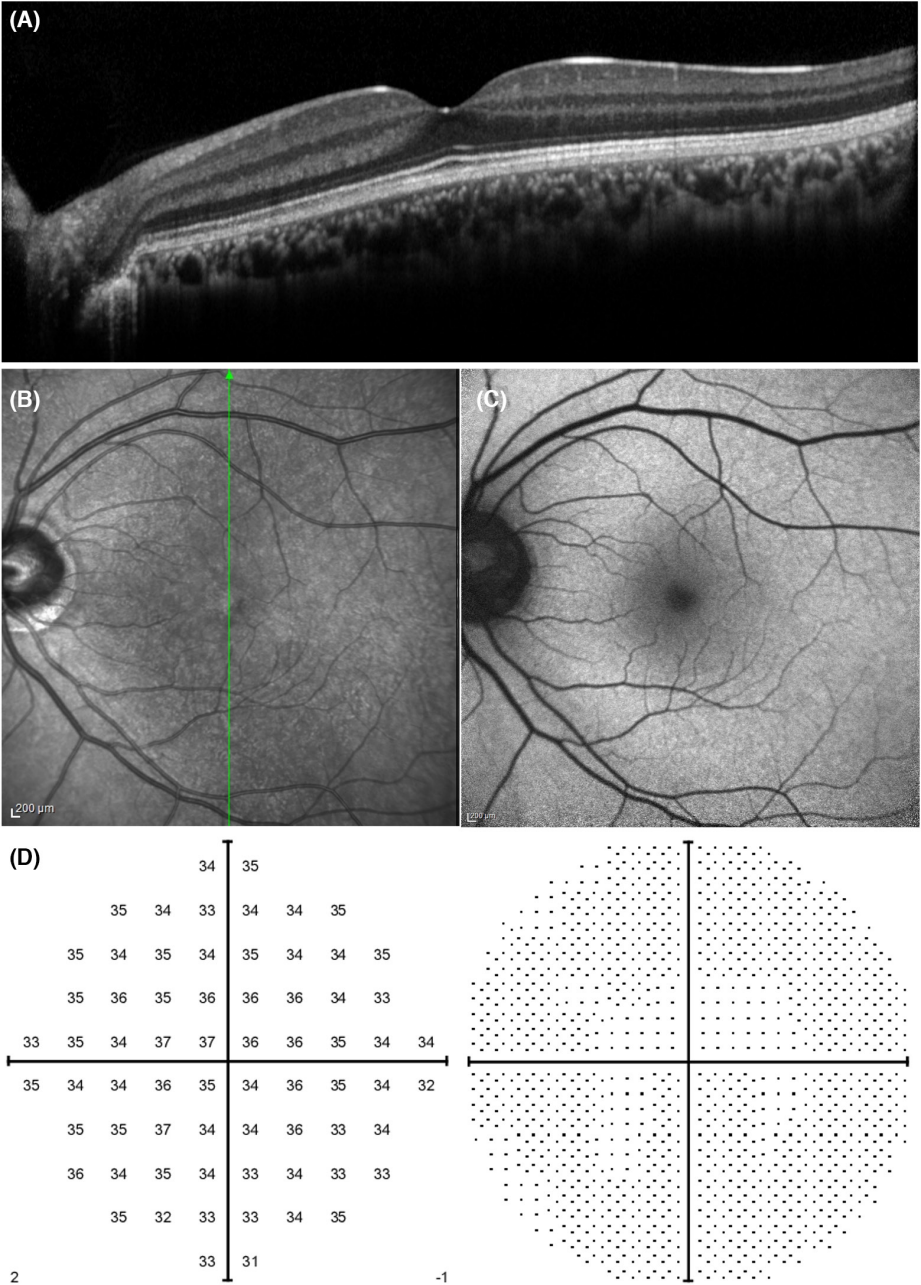
Optic coherence tomography (A), near‐infrared reflectance (B), fundus autofluorescence (C), and 10‐2 visual field test (Humphrey field analyzer) (D) findings of the left eye at 86 days after the vaccination.

Improvement of blurred vision in the left eye, which was a subjective symptom, was confirmed. The patient's BCVA was 20/16 in both eyes. The abnormal OCT, NIR, and FAF findings had almost disappeared (Figure 3A–C). HFA 10‐2 visual field testing did not reveal any abnormalities (Figure 3D).

### 2.4 Clinical findings 183 days after the vaccination

The patient reported that he was infected with COVID‐19183 days after vaccination. He had a fever for 2 days and a sore throat for 7 days but had no ocular symptoms. He did not visit our hospital during this period.

### 2.5 Clinical findings 366 days after the vaccination

**FIGURE 4:**
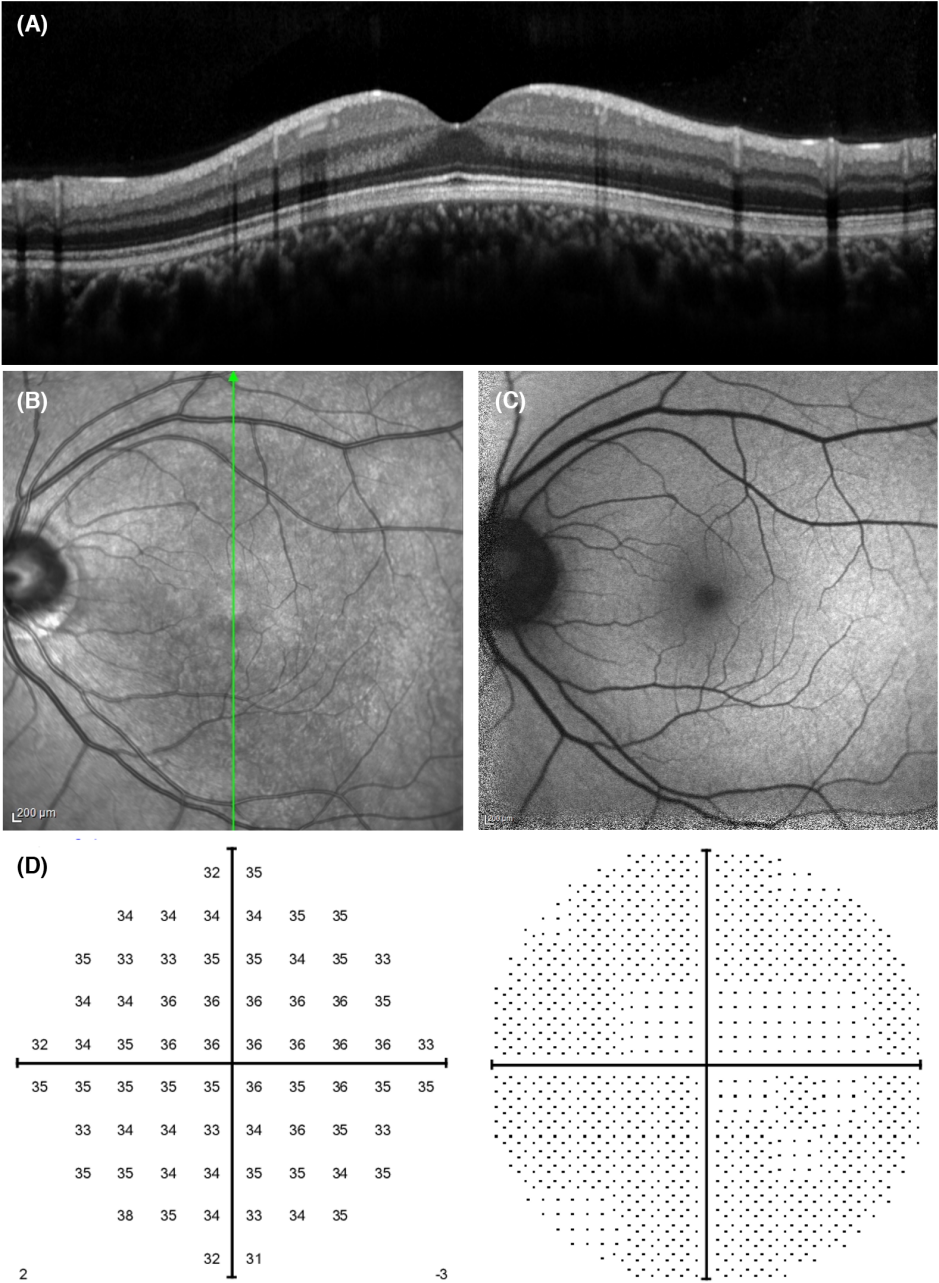
Optic coherence tomography (A), near‐infrared reflectance (B), fundus autofluorescence (C), and 10‐2 visual field test (Humphrey field analyzer) (D) findings of the left eye at 336 days after the vaccination.

The patient's subjective ocular symptoms had disappeared. His BCVA was 20/16 in both eyes. OCT, NIR, and FAF imaging did not reveal any abnormalities (Figure 4A–C). In addition, 10‐2 visual field testing did not reveal any visual field abnormality (Figure 4D). The patient did not receive any additional COVID‐19 vaccine during course of the AMN.

## 3 DISCUSSION AND CONCLUSIONS

Acute macular neuroretinopathy was first reported by Bos and Deutman in 1975.^25^ AMN is a rare retinal disease that commonly occurs in young women, who often report being aware of a sudden paracentral scotoma. Although the etiology of AMN is not clear, it is believed to be caused by circulatory dysregulation^26^ and disturbance of the deep capillary plexus in the outer layer of the retina due to ischemic events.^27^

In a review of 156 eyes of 101 patients with AMN, the onset of AMN was associated with nonspecific influenza‐like illnesses or fever (47.5%), contraceptive use (35.6%), trauma, and dehydration. Most of the patients included in the review were female (84.2%), with mean age of 29.5 years.^23^ Other than infections, intravitreal anti‐vascular endothelial growth factor injections, oral contraceptive pills, hypotension, intravenous contrast, intravenous ephedrine/epinephrine, caffeine, prothrombin‐associated antiphospholipid antibodies, and preeclampsia are reported to be risk factors for AMN. However, none of these risk factors are applicable in the present case.

The characteristic findings of AMN are the presence of reddish‐brown petaloid perifoveal lesions, with the tips of the petals pointing toward the fovea; however, this symptom was not clearly observed in the present case.

Retinal vascular abnormalities caused by COVID‐19 have been reported. COVID‐19 causes hypercoagulability due to various other pathological events, leading to proinflammatory and antifibrinolytic conditions, such as the development of antiphospholipid antibodies and elevated D‐dimer levels. These are believed to induce thrombus formation, leading to local embolism in small and microvessels in target organs.^28^, ^29^ In a previous study, abnormal retinal vascular findings (microhemorrhage, retinal vascular tortuosity, and cotton wool spots) were observed in 25 of 216 eyes (11.6%) of patients with COVID‐19. These signs were observed even in systemically asymptomatic patients.^30^ These previous reports suggest that a relatively large number of patients with COVID‐19 may have hypercoagulable retinal microvascular impairments, including AMN, even in the absence of ophthalmological symptoms.

A systolic blood pressure of 140 mmHg and/or a diastolic blood pressure of 90 mmHg are reported to be strongly associated with retinal vasculopathy in patients with COVID‐19.^30^ However, hypertension was not observed in the present case.

Retinal vascular disease can occur not only in patients with COVID‐19 but in those who have received COVID‐19 vaccines as well. The mechanism underlying this complication is believed to be the promotion of thrombus formation by oral contraceptives in the postinoculation inflammatory state and decrease in blood flow in the deep retinal capillary plexus due to the decrease in circulating plasma volume associated with the inflammatory reaction caused by inoculation.^31^ In a previous study, OCT angiography confirmed a decrease in deep retinal capillary plexus blood flow after COVID‐19 vaccination.^32^

In a previous review, the mean age at onset of retinopathy, including AMN and PAMM, after vaccination was 24.8 ± 4.8 years, whereas the mean duration from vaccination to onset of ocular symptoms was 3.1 ± 2.4 days. In addition, five patients (35.7%) showed bilateral lesions.^33^ The young age at onset and the duration from vaccination to disease onset in the abovementioned review are similar to those in this case.

The patients in most reported cases of AMN after COVID‐19 vaccination were female. To our knowledge, there are only two reports of AMN in males after COVID‐19. The patients in these reports were aged 35^34^ and 54^35^ years old. The 35‐year‐old man developed bilateral AMN complicated by PAMM after receiving the Oxford–AstraZeneca COVID‐19 vaccine.

In the present case, the patient was infected with COVID‐19183 days after the second vaccination. However, he did not experience any subjective ocular symptoms at the time, and his subsequent ophthalmological examination findings were normal. Although some ocular diseases developed after COVID‐19 spontaneously improved, the patient in the present case reported that he did not experience any subjective ocular symptoms after he got infected. Since his antibody titer was not measured during the course of the disease, the amounts of antibodies were produced after the first and second vaccinations, and COVID‐19 infection is unknown. Therefore, why COVID‐19 vaccination caused AMN, but the subsequent COVID‐19 infection did not remains unclear.

In conclusion, this long‐term report describes a case of a young, healthy, male patient who developed unilateral AMN after COVID‐19 vaccination. After the onset of ANM, the patient was followed up without any treatment, and his subjective ophthalmological symptoms persisted for approximately 3 months. However, his abnormal ocular examination findings improved approximately 1 month after the onset ANM and almost disappeared 3 months after onset. The patient was infected with COVID‐19 6 months after the onset of AMN; however, he did not experience any subjective symptoms and showed no abnormal findings in subsequent ophthalmological examinations. COVID‐19 vaccination can cause AMN in healthy young men. Therefore, a review of vaccination history is important for diagnosis.

## AUTHOR CONTRIBUTIONS

**Shunsuke Ikema:** Conceptualization; data curation; writing – review and editing. **Gen Miura:** Conceptualization; data curation; formal analysis; investigation; project administration; writing – original draft. **Daisuke Shimizu:** Conceptualization; data curation; writing – review and editing. **Takayuki Baba:** Conceptualization; supervision; writing – review and editing.

## FUNDING INFORMATION

No funding was received for this case report.

## CONFLICT OF INTEREST STATEMENT

The authors declare that there is no conflict of interest regarding the publication of this article.

## ETHICS STATEMENT

Ethical approval is not required for this study in accordance with national guidelines.

## CONSENT

Written informed consent was obtained from the patient for publication of this case report and any accompanying images. A copy of the written consent is available for review by the Editor‐in‐Chief of this journal.

## Data Availability

The datasets generated and/or analyzed during this study are available from the corresponding author upon reasonable request.

## References

[1] Furer V , Zisman D , Kibari A , Rimar D , Paran Y , Elkayam O . Herpes zoster following BNT162b2 mRNA COVID‐19 vaccination in patients with autoimmune inflammatory rheumatic diseases: a case series. Rheumatology (Oxford). 2021;60:Si90‐si95. 33848321 10.1093/rheumatology/keab345 PMC8083327

[2] Thimmanagari K , Veeraballi S , Roach D , Al Omour B , Slim J . Ipsilateral zoster ophthalmicus post COVID‐19 vaccine in healthy young adults. Cureus. 2021;13(7):e16725. 34471577 10.7759/cureus.16725 PMC8402883

[3] Alkhalifah MI , Alsobki HE , Alwael HM , Al Fawaz AM , Al‐Mezaine HS . Herpes simplex virus keratitis reactivation after SARS‐CoV‐2 BNT162b2 mRNA vaccination: a report of two cases. Ocul Immunol Inflamm. 2021;29(6):1238‐1240. 34637667 10.1080/09273948.2021.1986548

[4] Yasaka Y , Hasegawa E , Keino H , et al. A multicenter study of ocular inflammation after COVID‐19 vaccination. Jpn J Ophthalmol. 2023;67(1):14‐21. 36417027 10.1007/s10384-022-00962-9 PMC9684958

[5] Furer V , Eviatar T , Zisman D , et al. Immunogenicity and safety of the BNT162b2 mRNA COVID‐19 vaccine in adult patients with autoimmune inflammatory rheumatic diseases and in the general population: a multicentre study. Ann Rheum Dis. 2021;80(10):1330‐1338. 34127481 10.1136/annrheumdis-2021-220647

[6] ElSheikh RH , Haseeb A , Eleiwa TK , Elhusseiny AM . Acute uveitis following COVID‐19 vaccination. Ocul Immunol Inflamm. 2021;29(6):1207‐1209. 34379565 10.1080/09273948.2021.1962917

[7] Mudie LI , Zick JD , Dacey MS , Palestine AG . Panuveitis following vaccination for COVID‐19. Ocul Immunol Inflamm. 2021;29(4):741‐742. 34213988 10.1080/09273948.2021.1949478

[8] Papasavvas I , Herbort CP Jr . Reactivation of Vogt‐Koyanagi‐Harada disease under control for more than 6 years, following anti‐SARS‐CoV‐2 vaccination. J Ophthalmic Inflamm Infect. 2021;11(1):21. 34224024 10.1186/s12348-021-00251-5 PMC8256412

[9] Goyal M , Murthy SI , Annum S . Bilateral multifocal choroiditis following COVID‐19 vaccination. Ocul Immunol Inflamm. 2021;29(4):753‐757. 34344280 10.1080/09273948.2021.1957123

[10] Pichi F , Aljneibi S , Neri P , Hay S , Dackiw C , Ghazi NG . Association of ocular adverse events with inactivated COVID‐19 vaccination in patients in Abu Dhabi. JAMA Ophthalmol. 2021;139(10):1131‐1135. 34473209 10.1001/jamaophthalmol.2021.3477 PMC8414361

[11] Inagawa S , Onda M , Miyase T , et al. Multiple evanescent white dot syndrome following vaccination for COVID‐19: a case report. Medicine (Baltimore). 2022;101(2):e28582. 35029236 10.1097/MD.0000000000028582 PMC8758041

[12] Rabinovitch T , Ben‐Arie‐Weintrob Y , Hareuveni‐Blum T , et al. Uveitis after the BNT162b2 mRNA vaccination against SARS‐CoV‐2 infection: a possible association. Retina (Philadelphia, Pa). 2021;41(12):2462‐2471. 34369440 10.1097/IAE.0000000000003277

[13] Maleki A , Look‐Why S , Manhapra A , Foster CS . COVID‐19 recombinant mRNA vaccines and serious ocular inflammatory side effects: real or coincidence? J Ophthalmic Vis Res. 2021;16(3):490‐501. 34394876 10.18502/jovr.v16i3.9443 PMC8358769

[14] Helmchen C , Buttler GM , Markewitz R , Hummel K , Wiendl H , Boppel T . Acute bilateral optic/chiasm neuritis with longitudinal extensive transverse myelitis in longstanding stable multiple sclerosis following vector‐based vaccination against the SARS‐CoV‐2. J Neurol. 2022;269(1):49‐54. 34131771 10.1007/s00415-021-10647-x PMC8205198

[15] Leber HM , Sant'Ana L , Konichi da Silva NR , et al. Acute thyroiditis and bilateral optic neuritis following SARS‐CoV‐2 vaccination with CoronaVac: a case report. Ocul Immunol Inflamm. 2021;29(6):1200‐1206. 34402726 10.1080/09273948.2021.1961815

[16] Caudill GB , Wolin MJ . Myelin oligodendrocyte glycoprotein and neuromyelitis optica/aquaporin‐4 antibody negative COVID‐19‐associated optic neuritis. J Neuroophthalmol. 2023;43(1):e1‐e2. 34348359 10.1097/WNO.0000000000001364 PMC9924726

[17] Benito‐Pascual B , Gegúndez JA , Díaz‐Valle D , et al. Panuveitis and optic neuritis as a possible initial presentation of the novel coronavirus disease 2019 (COVID‐19). Ocul Immunol Inflamm. 2020;28(6):922‐925. 32870739 10.1080/09273948.2020.1792512

[18] Kogure C , Kikushima W , Fukuda Y , et al. Myelin oligodendrocyte glycoprotein antibody‐associated optic neuritis in a COVID‐19 patient: a case report. Medicine (Baltimore). 2021;100(19):e25865. 34106635 10.1097/MD.0000000000025865 PMC8133173

[19] Rho J , Dryden SC , McGuffey CD , Fowler BT , Fleming J . A case of non‐Arteritic anterior ischemic optic neuropathy with COVID‐19. Cureus. 2020;12(12):e11950. 33425529 10.7759/cureus.11950 PMC7785499

[20] Tsukii R , Kasuya Y , Makino S . Nonarteritic anterior ischemic optic neuropathy following COVID‐19 vaccination: consequence or coincidence. Case Rep Ophthalmol Med. 2021;2021:5126254. 34659851 10.1155/2021/5126254 PMC8516575

[21] Jin SX , Juthani VV . Acute corneal endothelial graft rejection with coinciding COVID‐19 infection. Cornea. 2021;40(1):123‐124. 32889957 10.1097/ICO.0000000000002556 PMC7513958

[22] Wan EYF , Chui CSL , Lai FTT , et al. Bell's palsy following vaccination with mRNA (BNT162b2) and inactivated (CoronaVac) SARS‐CoV‐2 vaccines: a case series and nested case‐control study. Lancet Infect Dis. 2022;22(1):64‐72. 34411532 10.1016/S1473-3099(21)00451-5 PMC8367195

[23] Bhavsar KV , Lin S , Rahimy E , et al. Acute macular neuroretinopathy: a comprehensive review of the literature. Surv Ophthalmol. 2016;61(5):538‐565. 26973287 10.1016/j.survophthal.2016.03.003

[24] Fekri S , Khorshidifar M , Dehghani MS , Nouri H , Abtahi SH . Acute macular neuroretinopathy and COVID‐19 vaccination: case report and literature review. J Fr Ophtalmol. 2023;46(1):72‐82. 36496293 10.1016/j.jfo.2022.09.008 PMC9684098

[25] Bos PJ , Deutman AF . Acute macular neuroretinopathy. Am J Ophthalmol. 1975;80(4):573‐584. 1180301 10.1016/0002-9394(75)90387-6

[26] Munk MR , Jampol LM , Cunha Souza E , et al. New associations of classic acute macular neuroretinopathy. Br J Ophthalmol. 2016;100(3):389‐394. 26294104 10.1136/bjophthalmol-2015-306845

[27] Casalino G , Arrigo A , Romano F , Munk MR , Bandello F , Parodi MB . Acute macular neuroretinopathy: pathogenetic insights from optical coherence tomography angiography. Br J Ophthalmol. 2019;103(3):410‐414. 29844084 10.1136/bjophthalmol-2018-312197

[28] Zhang Y , Xiao M , Zhang S , et al. Coagulopathy and antiphospholipid antibodies in patients with Covid‐19. N Engl J Med. 2020;382(17):e38. 32268022 10.1056/NEJMc2007575 PMC7161262

[29] Kichloo A , Dettloff K , Aljadah M , et al. COVID‐19 and hypercoagulability: a review. Clin Appl Thromb Hemost. 2020;26:1076029620962853. 33074732 10.1177/1076029620962853 PMC7592310

[30] Sim R , Cheung G , Ting D , et al. Retinal microvascular signs in COVID‐19. Br J Ophthalmol. 2022;106(9):1308‐1312. 33741583 10.1136/bjophthalmol-2020-318236

[31] Mambretti M , Huemer J , Torregrossa G , Ullrich M , Findl O , Casalino G . Acute macular neuroretinopathy following coronavirus disease 2019 vaccination. Ocul Immunol Inflamm. 2021;29(4):730‐733. 34187278 10.1080/09273948.2021.1946567

[32] Bøhler AD , Strøm ME , Sandvig KU , Moe MC , Jørstad ØK . Acute macular neuroretinopathy following COVID‐19 vaccination. Eye (Lond). 2022;36(3):644‐645. 34158652 10.1038/s41433-021-01610-1 PMC8217204

[33] Haseeb AA , Solyman O , Abushanab MM , Abo Obaia AS , Elhusseiny AM . Ocular complications following vaccination for COVID‐19: a one‐year retrospective. Vaccines (Basel). 2022;10(2):342. 35214800 10.3390/vaccines10020342 PMC8875181

[34] Vinzamuri S , Pradeep TG , Kotian R . Bilateral paracentral acute middle maculopathy and acute macular neuroretinopathy following COVID‐19 vaccination. Indian J Ophthalmol. 2021;69(10):2862‐2864. 34571652 10.4103/ijo.IJO_1333_21 PMC8597515

[35] Diafas A , Ghadiri N , Beare N , Madhusudhan S , Pearce I , Tan SZ . Comment on: ‘Paracentral acute middle maculopathy and acute macular neuroretinopathy following SARS‐CoV‐2 infection’. Eye (Lond). 2022;36(7):1507‐1509. 34341483 10.1038/s41433-021-01709-5 PMC8327597

